# Epigenetic remodeling during early embryonic development

**DOI:** 10.3389/fcell.2026.1750381

**Published:** 2026-02-17

**Authors:** Xiaoyu Wan, Shibin Zhang, Jingyu Li, Deying Kong, Mo Chen

**Affiliations:** 1 Department of Physiology, College of Basic Medical Science, Zunyi Medical University, Zunyi, Guizhou, China; 2 Chongqing Key Laboratory of Human Embryo Engineering and Precision Medicine, Center for Reproductive Medicine, Women and Children’s Hospital of Chongqing Medical University, Chongqing, China; 3 Laboratory Animal Center, Zunyi Medical University, Zunyi, Guizhou, China

**Keywords:** early embryo development, epigenetic reprogramming, histone modifications, mammals, metabolically-associated histone marks

## Abstract

In mammals, the fusion of sperm and oocyte gives rise to a totipotent zygote, which undergoes a series of cleavage divisions and differentiation events. During this process, the embryo transitions from totipotency to pluripotency, accompanied by extensive epigenetic reprogramming. With continuous innovation of low-input multi-omics technology and other methods, the relationship between epigenetic remodeling and embryonic development has been gradually revealed. This review synthesizes recent advances in our understanding of the dynamics of epigenetic reprogramming during early embryogenesis in mice and humans. It covers the remodeling of DNA methylation, histone modifications, chromatin accessibility, and three-dimensional chromatin architecture, with a particular focus on the dynamic features of histone modifications. The patterns of common histone modifications such as methylation, acetylation, and ubiquitination are elaborated. Furthermore, the review outlines both the emerging roles of metabolism-associated modifications such as crotonylation and lactylation in genomic targeting and transcriptional regulation, and the dynamic patterns of histone variant incorporation.

## 1 Introduction

Early embryonic development undergoes a series of dramatic changes including epigenetic remodeling as well as key biological events such as fertilization (Du et al., 2022), zygotic genome activation (ZGA) (Zou et al., 2024), and differentiation. Upon fertilization, the union of sperm and oocyte necessitates extensive epigenetic reprogramming to harmonize the disparate parental genomes and establish the totipotent state of the zygote (Zhou and Dean, 2014). Two fate decisions occur during early embryonic development: the first produces the Inner cell mass (ICM) and Trophectoderm (TE), while the second leads to ICM differentiating into the Primitive endoderm (PE) (Zhu and Zernicka-Goetz, 2020), forming the yolk sac, and the Epiblast (EPI) producing the embryo. During gastrulation, cell fate specification is accompanied by genome-wide remodeling of epigenetic landscape (Argelaguet et al., 2019), wherein distal enhancers marked by H3K27ac play an essential role in spatiotemporal gene regulation (Yang et al., 2019). Notably, early asymmetry in cell fate commitment emerges during the initial stages of mammalian embryogenesis. In the mouse 2-cell embryo, asymmetric distribution of factors such as lincGET initiates and progressively amplifies the first lineage bias (Wang J. et al., 2018). Similarly, the two blastomeres of human 2-cell embryos have been shown to contribute unequally to subsequent embryonic development, reflecting a conserved mechanism of fate asymmetry that precedes morphological differentiation (Junyent et al., 2024). But the link and underlying mechanisms between cell fate and epigenetic reprogramming are not yet clear.

Epigenetic remodeling is a complex process involving the erasure of gamete-specific marks to establish developmental totipotency, coupled with the subsequent activation of programs for initial lineage commitment (Xu and Xie, 2017). To ensure rapid and profound cellular identity transitions, a sophisticated epigenetic regulatory system must operate with precision. This system maintains the specificity of cellular states and exerts precise control over gene expression through coordinated mechanisms, including transcriptional silencing *via* DNA methylation (Zhu et al., 2025), dynamic chromatin modulation by histone modifications, regulation of chromatin accessibility and 3D architectural remodeling.

In this review, we focus on the dynamics of epigenetic remodeling during early embryogenesis in mice and humans. We summarize the dynamics of DNA methylation and associated enzymes, and delineate the changes in common histone modifications such as methylation, acetylation, and ubiquitination. We also outline metabolism-associated histone modifications such as crotonylation and lactylation in genomic targeting and transcriptional regulation and discuss the dynamics of histone variants. Particular attention is given to the changing landscapes of histone modifications and recent advances in their regulatory enzymes. In addition, we cover the remodeling of chromatin accessibility and three-dimensional architecture. Where appropriate, we also explore potential links between specific epigenetic modifications and lineage-specific gene expression.

## 2 DNA methylation: a key epigenetic regulator in the embryo

DNA methylation undergoes precisely orchestrated reprogramming, directing cell fate transitions *via* dynamic epigenetic regulation (Messerschmidt et al., 2014). In mouse zygotes, the paternal pronucleus exhibits significantly higher DNA methylation levels than the maternal pronucleus. Subsequently, the paternal genome undergoes rapid active demethylation followed by passive dilution, reaching its lowest methylation level by the blastocyst stage (Yan et al., 2022). The maternal genome also experiences passive demethylation, achieving similar methylation levels to the paternal genome by the blastocyst stage (Smith et al., 2012). This global demethylation from the zygote to the blastocyst stage erases most gamete-inherited methylation marks, while the subsequent remethylation initiates the establishment of embryonic methylation patterns (Zeng and Chen, 2019). The establishment of DNA methylation with lineage and locus specificity following implantation relies on H3K36me2-mediated reprogramming (Lu X. et al., 2025). In human embryos, the paternal genome is also demethylated more rapidly than the maternal genome. However, a notable difference is observed in the timing of genome-wide demethylation: it occurs largely during the zygotic phase in mouse embryos, but initiates and largely completes between fertilization and the 2-cell stage in human embryos (Guo H. et al., 2014). Through genome-wide cycles of establishment and erasure, DNA methylation directs the reprogramming essential for resetting cell identity during embryogenesis.

The reprogramming of DNA methylation is dynamically regulated by the opposing activities of DNMT (writers) and TET (erasers) family enzymes (Charlton et al., 2020). *De novo* methylation is primarily orchestrated by DNMT3A and DNMT3B (Chen and Zhang, 2020), a process crucially enhanced by the cofactor DNMT3L (Chedin et al., 2002). While maintenance methylation is executed by DNMT1 with the assistance of UHRF1 (also known as Np95 or ICBP90) (Bostick et al., 2007; Sharif et al., 2007). Mouse oocytes and early embryos exhibit divergent expression dynamics of DNMT3A, DNMT3B, and DNMT1 (Uysal et al., 2017). Disruption of these enzymes or chaperone protein leads to severe consequences: DNMT3A knockout results in early postnatal mortality, DNMT3B deficiency causes embryonic lethality (Okano et al., 1999), knockdown of DNMT3A or DNMT1 results in developmental arrest at the 2- to 4-cell stage in a subset of embryos (Uysal et al., 2021). Loss of DNMT3L abrogates DNMT3A activity (Jia et al., 2007), and conditional knockout of maternal UHRF1 in primordial or growing oocytes results in embryonic arrest at the two-cell or blastocyst stage, respectively (Wu et al., 2021). Deficiency in all TET enzymes results primarily in two-cell arrest, whereas knockdown of TET3 alone permits a fraction of embryos to develop to the blastocyst stage (Arand et al., 2021). In early mammalian embryos, paternal genome demethylation is primarily driven by the active enzymatic activity of TET3 (Gu et al., 2011; Oswald et al., 2000; Wossidlo et al., 2011), whereas methylation on the maternal genome is largely diluted through passive replication dilution (Rougier et al., 1998). However, several studies have revealed cooperative roles of TET3-driven active demethylation and passive dilution in demethylating both parental genomes, challenging the previously segregated view of these mechanisms (Guo F. et al., 2014; Shen et al., 2014). Complementing these pathways, the base excision repair (BER) pathway has been implicated in genome-wide DNA demethylation in the mouse germline (Hajkova et al., 2010). Cellular studies have further clarified this role by demonstrating the specific recognition and excision of TET-mediated 5-carboxylcytosine (5caC), a process that directly links the BER pathway to active demethylation (He et al., 2011). In short, DNA methyltransferases (writers) and demethylases (erasers) are indispensable for the epigenetic reprogramming that governs mammalian early embryonic development.

## 3 Histone modifications

In mammalian early embryonic development, histone modifications serve as pivotal epigenetic mechanisms that orchestrate cell fate determination by dynamically regulating chromatin architecture and gene expression patterns (Xu et al., 2020). In this part, we provide an integrated synthesis of recent findings on the dynamics of different histone modifications, histone variants, and their associated regulatory enzymes during early embryonic development.

### 3.1 Histone methylation

Histone methylation possesses distinct biological advantages among histone modifications, exerting central roles in gene expression regulation, cell fate determination, and developmental processes through its precise site specificity, dynamic reversibility, and functional diversity (Black et al., 2012; Hyun et al., 2017). For instance, H3K4me3 not only antagonizes H3K9me3 to license early transcription but also, through its demethylation in concert with H3K36me3 and transcription, further enables the establishment of the embryonic *de novo* methylome, while maternally inherited H3K27me3 governs post-implantation development (Stäubli and Peters, 2021).

#### 3.1.1 H3K4me3-dependent epigenetic regulation in early mammalian embryogenesis

**FIGURE 1:**
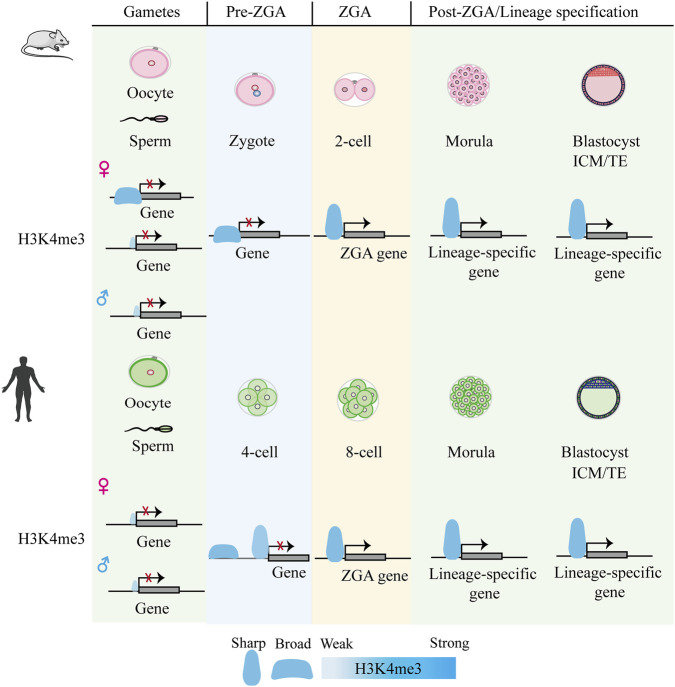
Epigenetic dynamics of H3K4me3 during early embryonic development in mouse and human. In mouse oocytes, H3K4me3 forms non-canonical broad domains, which resolve into canonical promoter-focused peaks after ZGA (Dahl et al., 2016; Liu et al., 2016; Zhang et al., 2016). These broad domains can be transiently transmitted to the maternal allele in early embryos (Dahl et al., 2016; Liu et al., 2016; Zhang et al., 2016). In contrast, such non-canonical domains are absent in human oocytes and early embryogenesis (Xia et al., 2019).

H3K4me3 is traditionally associated with transcription initiation at promoter regions (Lauberth et al., 2013). H3K4me3 in mouse oocytes not only marks active promoters but also forms broad domains (bdH3K4me3) at intergenic regions, putative enhancers, and Polycomb-repressed sites through a transcription-independent mechanism (Dahl et al., 2016; Liu et al., 2016; Zhang et al., 2016). These broad domains can be inherited by the zygote and persist into the early 2-cell stage but are subsequently erased by the late 2-cell stage, coinciding with the establishment of the canonical H3K4me3 pattern (Dahl et al., 2016; Liu et al., 2016; Zhang et al., 2016) In mouse sperm, H3K4me3 is primarily enriched at CpG-rich promoters (Yamaguchi et al., 2018). Despite this, the paternal genome exhibits no such H3K4me3 enrichment following fertilization, with its *de novo* establishment occurring subsequent to major ZGA (Zhang et al., 2016). Unlike in mice, H3K4me3 in human oocytes is distributed as sharp, canonical peaks at promoters, lacking non-canonical broad domains (Xia et al., 2019). After fertilization but before ZGA (pre-4-cell stage), a priming H3K4me3 state is deposited *de novo* at both promoters and distal regions, alongside the acquisition of chromatin openness (Xia et al., 2019) (Figure 1).

Given the critical functions of H3K4me3 in transcription and development, a central question lies in understanding how its specific genomic distribution is established and regulated. H3K4me3 is deposition by the multi-subunit COMPASS methyltransferase complex—comprising catalytic subunits SETD1A/B and MLL1-4, along with coregulatory components (Zhang J. et al., 2025). MLL2 is essential for mouse oocyte survival, where it catalyzes transcription-independent H3K4me3 deposition at unmethylated CpG-rich regions (Andreu-Vieyra et al., 2010; Hanna et al., 2018). MLL2 is also responsible for establishing H3K4me3 (both non-canonical and canonical patterns) in totipotent mouse embryos (Zhang J. et al., 2025). In contrast, upon pluripotency acquisition post-ZGA, SETD1A/B takes over, mediating transcription-dependent H3K4me3 deposition to directly promote pluripotency and blastocyst gene expression (Zhang J. et al., 2025). Concurrently, the transition of bdH3K4me3 to canonical H3K4me3 is largely mediated by KDM5B across species (Bu et al., 2022; Dahl et al., 2016; Dang et al., 2022). Nevertheless, determining its exact regulatory mechanisms has been hindered by substantial functional redundancy in the COMPASS methyltransferase complex family (Qu et al., 2018). A key insight came from the acute depletion of a shared COMPASS subunit in ESCs, which ablated all H3K4 methylation and revealed a specific requirement for H3K4me3 in RNA polymerase II pause-release and transcriptional elongation (Wang et al., 2023). Douillet, D.*, et al.* also employed CRISPR-based technology in mESCs to show that MLL2 protects developmental genes from repression by excluding PRC2 and DNA methylation machinery (Douillet et al., 2020). KDM5B/JARID1B is essential for neural lineage differentiation, where its depletion prevents the timely silencing of lineage-inappropriate genes (Schmitz et al., 2011). Thus, the dynamics of H3K4me3 are further modulated by COMPASS and KDM5 demethylases, enabling rapid, developmentally responsive epigenetic switching.

#### 3.1.2 H3K27me3-mediated reversible gene silencing

H3K27me3, a hallmark of facultative heterochromatin, plays a pivotal role in cell fate determination by establishing reversible chromatin condensation, leading to transcriptional silencing during specific developmental windows. Two prominent examples include the silencing of non-canonical imprinted genes (Inoue et al., 2017) and X-chromosome inactivation (Masui et al., 2023), both of which critically influence allele-specific expression through H3K27me3-mediated epigenetic regulation. Facultative heterochromatin formation is cooperatively established through PRC1- and PRC2-mediated deposition of H2AK119ub1 and H3K27me3, respectively (Tamburri et al., 2020). Recent studies in mouse embryos have elucidated a stepwise assembly mechanism whereby sequential enrichment of H2AK119ub1 (Chen et al., 2021; Mei et al., 2021), H3K27me2, and H3K27me3 leads to silencing of key developmental genes (Matsuwaka et al., 2025; Meng et al., 2020).

**FIGURE 2:**
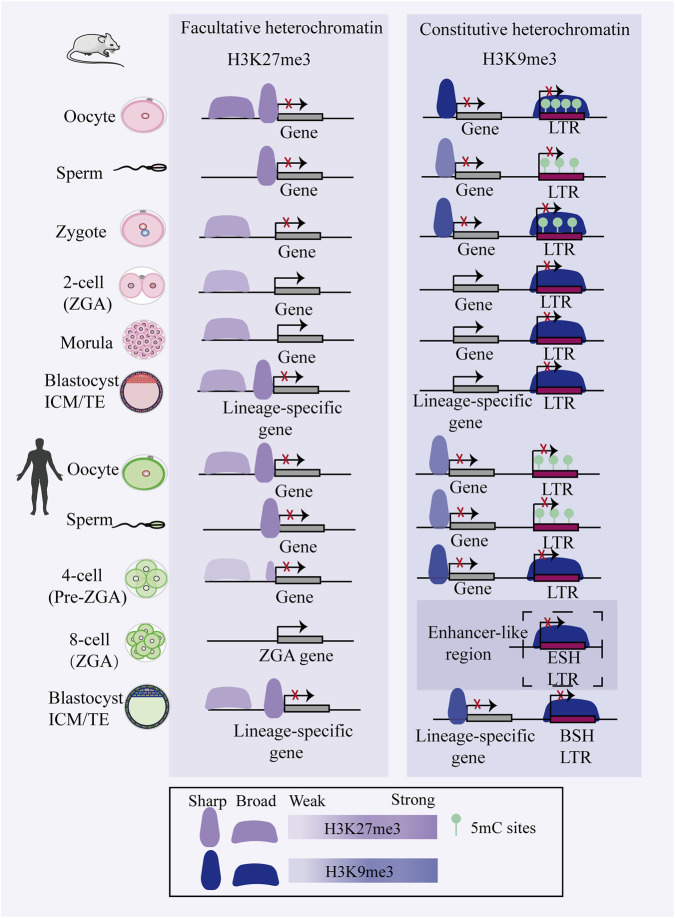
Comparative schematic of heterochromatin dynamics in mouse and human early embryos. During early embryonic development, H3K27me3 and H3K9me3 exhibit distinct reprogramming patterns that show partial conservation across species. In mouse embryos, promoter-associated H3K27me3 is extensively lost by the 2-cell stage and only gradually reestablished by the blastocyst stage, while distal H3K27me3 domains persist through blastocyst formation (Liu et al., 2016; Mei et al., 2021; Zhang et al., 2016). Human embryos display similar dynamics of H3K27me3 at both promoter and enhancer regions, indicating evolutionary conservation of this reprogramming process among mammals. Concurrently, H3K9me3 distribution demonstrates region-specific dynamics: its enrichment at long terminal repeat (LTR) retrotransposons gradually increases during development, whereas promoter-associated H3K9me3 is progressively erased following fertilization (Wang C. et al., 2018). Notably, stage-specific H3K9me3 domains present in human 8-cell embryos may be preconfigured for potential enhancer-like functions in later developmental stages (Xu et al., 2022; Yu et al., 2022). These findings reveal that H3K9me3 not only participates in transposon silencing but may also play more complex roles within the gene regulatory network of human embryogenesis. The term “enhancer-like region” refers to H3K9me3-marked regions that acquire enhancer marks in subsequent stages. This illustration was created with reference to (Du et al., 2022; Wang C. et al., 2018; Xu et al., 2020; Xu et al., 2022; Xu R. et al., 2025; Yu et al., 2022). Abbreviations: BSH, blastocyst-specific H3K9me3-marked; ESH, eight-cell specific H3K9me3-marked; 5mC, 5-methylcytosine.

During early mouse embryonic development, H3K27me3 exhibits a dynamic pattern characterized by parent-of-origin-specific inheritance and stage-specific reprogramming. Following fertilization, H3K27me3 on the paternal genome is rapidly erased, while a subset of non-canonical broad H3K27me3 domains at distal regulatory regions on the maternal allele is retained (Liu et al., 2016; Zheng et al., 2016). These maternally inherited domains mediate a DNA methylation-independent, non-canonical form of genomic imprinting, which persists through the blastocyst stage before transitioning into the canonical imprinting mechanism after implantation (Inoue et al., 2017; Zheng et al., 2016). In parallel, canonical H3K27me3 marks at promoters of developmental genes—including Hox and other key regulators—are also lost shortly after fertilization (Zheng et al., 2016). These canonical repressive marks are subsequently re-established specifically at developmental gene promoters in the epiblast, suggesting an association with the expression of lineage-specification genes following implantation (Liu et al., 2016; Zheng et al., 2016). In contrast, human oocytes exhibit canonical H3K27me3 enrichment at promoter regions of developmental genes and partially methylated domains (PMDs), which is largely erased by the 8-cell stage (Xia et al., 2019). Unlike the mouse, the human early embryo retains most paternal epigenetic information and lack H3K27me3-dependent imprinting mechanisms (Yuan et al., 2024) (Figure 2).

H3K27me3 exhibits pronounced distributional heterogeneity and functional diversity during early embryonic lineage specification (Dahl et al., 2010; Pailles et al., 2022; Yang et al., 2018). Distinct H3K27me3 patterns between the ICM and TE contribute to cell fate decisions, likely by silencing lineage-specific genes or modulating transcription factor activities (Dahl et al., 2010; Saha et al., 2013; Yang et al., 2018). Interestingly, the coordinated action of the PRC2 component EED and the demethylase KDM6B underlies the differential deposition of H3K27me3 at loci of key transcription factors (including CDX2 and GATA3),thereby driving their lineage-specific silencing in the ICM *versus* activation in the TE (Saha et al., 2013). In post-implantation mouse embryos, Yang, X. *et al.* further demonstrated that key developmental genes undergo differential epigenetic silencing across lineages: embryonic cells primarily employ H3K27me3, whereas the extra-embryonic ectoderm (ExE) utilizes DNA methylation (DNAme) for stable repression (Yang et al., 2018). In both human embryonic stem cells and mouse models, ablation of PRC2 components perturbs pluripotency, underscoring the complex, lineage- and state-specific roles of PRC2 in cell fate determination (Shan et al., 2017). Moreover, defective H3K27me3 imprinting in somatic cell nuclear transfer (SCNT) embryos constitutes a major epigenetic barrier to post-implantation development (Wang et al., 2020). Together, these findings establish H3K27me3 as a pivotal epigenetic regulator that enables precise spatiotemporal control of developmental trajectories through highly adaptable and lineage-specific gene silencing mechanisms.

#### 3.1.3 H3K9me3 heterochromatin

H3K9me3, a hallmark of constitutive heterochromatin distinct from H3K27me3 (Grewal, 2023), is essential for gene silencing and genome stability—particularly at repetitive elements such as pericentromeric regions and telomeres (Saksouk et al., 2015). Intriguingly, *de novo* H3K9me3 deposition in the paternal pronucleus post-fertilization initially serves a non-repressive role by compacting promoter regions rather than inducing silencing (Burton et al., 2020). In mammals, H3K9 methylation deposition is catalyzed by methyltransferases including SUV39H1/2, SETDB1/2 and G9a/GLP, whose coordinated activities are critical for embryogenesis (Burton et al., 2020; Eymery et al., 2016; Kim et al., 2016; Wang C. et al., 2018). Conditional knockout of SETDB1 in mouse oocytes severely disrupts meiotic progression, significantly reduces the number of mature oocytes, and impairs preimplantation development (Eymery et al., 2016; Kim et al., 2016). Heterochromatin-mediated gene silencing may be partially achieved through the segregation of tightly compacted chromatin into HP1-enriched condensates formed *via* phase separation (Larson et al., 2017). The H3K9me3 demethylase KDM4A removes this mark at broad H3K4me3 domains to maintain maternal epigenome integrity and ensure e the proper initiation of ZGA, thereby safeguarding embryonic developmental trajectory (Sankar et al., 2020).

Of note, dynamic reprogramming of H3K9me3 plays an essential role in cell fate transitions during embryonic development. In mouse early embryos, H3K9me3 is erased from promoter regions after fertilization and is not restored until post-implantation, however, its levels increase at long terminal repeats (LTRs) throughout preimplantation development (Wang C. et al., 2018). Similarly, in human embryos, stage-specific deposition of H3K9me3 is a conserved mechanism critical for transposon silencing and lineage commitment (Xu et al., 2022) (Figure 2). For instance, in the trophectoderm, *de novo* H3K9me3 domains are associated with limited binding of pluripotency transcription factors at ICM-specific regulatory elements derived from hominoid-specific retrotransposons (Xu et al., 2022; Yu et al., 2022). This H3K9me3 deposition may facilitate the lineage separation between the ICM and TE (Xu et al., 2022; Yu et al., 2022). Additionally, Wang, L.*, et al.* demonstrated that during implantation, *de novo* H3K9me3 deposition in the extra-embryonic ectoderm safeguards lineage identity by antagonizing *TFAP2C* binding at key developmental genes such as *CDX2 and NANOG* (Wang L. et al., 2024). This finding underscores the pivotal role of the H3K9me3-mediated chromatin environment in ensuring lineage commitment during embryogenesis. In summary, H3K9me3 serves as a critical epigenetic gatekeeper that reinforces cell fate restriction through silencing retrotransposons and selectively repressing lineage-inappropriate genes.

#### 3.1.4 Bivalent H3K4me3 and H3K27me3

Bivalent chromatin domains, characterized by the coexistence of the activating H3K4me3 and repressive H3K27me3 marks, enable genes to transition from a poised to either an active or repressed state in ESCs and mouse embryos (Bernstein et al., 2006; Dahl et al., 2010). In the early mouse embryo, these bivalent promoters display marked spatiotemporal dynamics and distributional heterogeneity. Their differential occupancy across the ICM, TE, and three blastocyst-derived stem cell types can be primarily attributed to the abundance and genomic dynamics of H3K27me3, more so than to H3K4me3 (Dahl et al., 2010; Rugg-Gunn et al., 2010). In the ICM, bivalent chromatin marks at promoters of developmental genes like *Hox* implies a role in poising these loci for subsequent activation, indicating a temporal uncoupling between mark deposition and function (Liu et al., 2016). This phenomenon has also been observed in post-implantation embryos (Li Y. et al., 2025). Notably, a subset of transiently maintained bivalent domains (TB domains) is specifically identified in the EPI (Li Y. et al., 2025). In trophoblast stem cells (TS cells), KDM5B deficiency alters H3K4 methylation at self-renewal genes, upregulates trophoblast lineage genes, and thereby biases cell fate toward trophoblast differentiation (Xu and Kidder, 2018). In summary, through its unique combinatorial histone signature and dynamic regulatory properties, bivalent chromatin may serve as a key regulatory node in early embryonic development.

#### 3.1.5 H3K36me3-epigenetic modification crosstalk

H3K36me3, catalyzed by SET domain containing 2 (SETD2), is associated with actively transcribed gene bodies. This mark recruits DNA methyltransferase Dnmt3b to facilitate gene body methylation, thereby ensuring the fidelity of transcription initiation (Neri et al., 2017). Yano, S.*, et al.* further demonstrated that in mouse oocytes, H3K36me3 and H3K36me2 collectively establish an essential chromatin context for DNMT3A/DNMT3L complex-dependent DNA methylation (Yano et al., 2022). Maternal SETD2 depletion causes oocyte maturation defects and subsequent one-cell arrest (Xu et al., 2019). Their study also revealed that Setd2 depletion causes widespread epigenome dysregulation in oocytes, characterized by loss of H3K36me3, failure of DNA methylome establishment, aberrant invasion of H3K4me3 and H3K27me3 into former H3K36me3 domains. During embryonic development, H3K36me3 plays particularly crucial regulatory roles. Nuclear transfer embryos exhibit abnormal enrichment of broad H3K4me3 domains and H3K27me3 signals, accompanied by increased bivalent marking at gene promoters. Notably, Setd2 overexpression can reconstitute H3K36me3 at gene bodies, restore expression of ZGA-related genes, and exclude H3K27me3 from bivalent promoters (Zhang X. et al., 2025). These studies collectively illustrate the complex interplay between H3K36me3 and other histone modifications. H3K36me3-marked regions appear to maintain a spatially competitive relationship with both H3K4me3 and H3K27me3. Furthermore, mouse stem cells harbor atypical heterochromatin domains co-enriched with H3K9me3 and H3K36me3. Destabilization of these domains leads to acquisition of active enhancer features, revealing coordinated regulation between different histone modification systems in maintaining cellular identity (Barral et al., 2022). In summary, the dynamic establishment and maintenance of H3K36me3 are essential for orchestrating proper epigenomic programming and ensuring the fidelity of gene expression.

#### 3.1.6 H3K4me1: enhancer-mediated precise regulation of embryonic development

**FIGURE 3:**
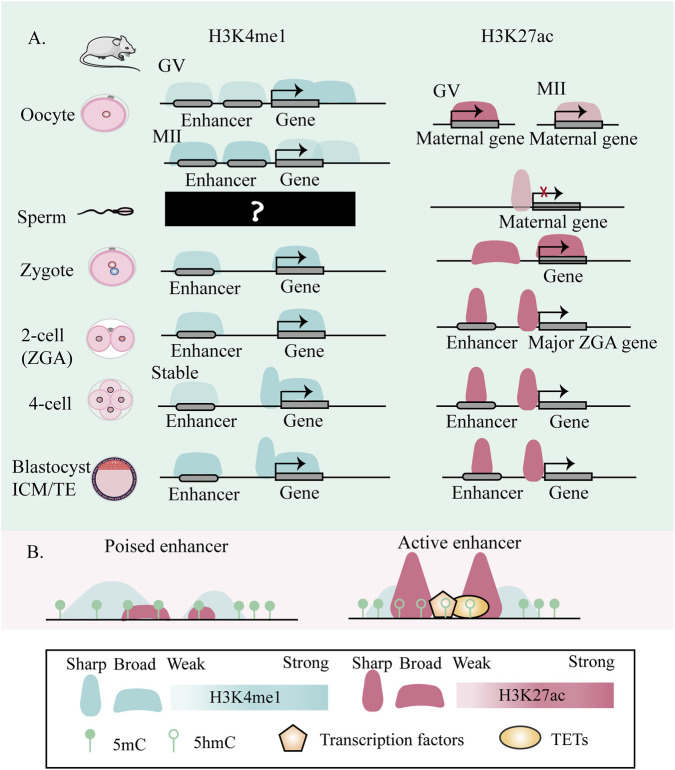
Dynamic changes of enhancer H3K4me1 and H3K27ac during Mouse embryonic development. **(A)** Systematically delineates the dynamic changes of two enhancer marks, H3K4me1 and H3K27ac, during mouse embryonic development. Both modifications are present in oocytes and undergo remodeling during maturation, though the distribution pattern of H3K4me1 in sperm remains unclear. After fertilization, H3K4me1 levels at promoters and enhancers change in a region-specific manner, culminating in a marked transition in chromatin architecture by the 4-cell stage, characterized by sharper peak profiles. Concurrently, H3K27ac displays broad domains in oocytes and zygotes, which resolve into canonical sharp peaks following zygotic genome activation. This illustration was created with reference to (Kang et al., 2021; Liu et al., 2025; Wang et al., 2022; Hu, 2022). **(B)** Enhancer regions exhibit two distinct co-occurrence profiles of H3K27ac and H3K4me1. H3K4me1 serves as a shared marker of active and poised enhancers, while H3K27ac deposition is highly variable. This heterogeneity underlies divergent enhancer functional states and regulatory capacities, essential for guiding cell fate decisions. This illustration was created with reference to (Kang et al., 2021; Liu et al., 2025; Wang et al., 2022; Hu, 2022).

H3K4me1 serves as a core epigenetic feature of enhancers, where its combinatorial interactions with H3K27ac (marking active enhancers) or H3K27me3 (demarcating poised enhancers) precisely dictate enhancer activity states. While both H3K4me1 and H3K27ac characterize enhancers, H3K4me1 exhibits a more direct role in nucleosome positioning, whereas H3K27ac predominantly modulates enhancer RNA transcription (Kang et al., 2021). Therefore, the localization and distribution of H3K4me1 throughout mouse oogenesis and preimplantation development are critical, as they may influence lineage-specific gene expression. Strikingly, the non-canonical, widespread H3K4me1 pattern was markedly remodeled at the 4-cell stage (Liu et al., 2025; Hu, 2022). Specifically, promoter-associated H3K4me1 from oocytes was inherited to the 2-cell stage but extensively erased at the 4-cell stage, with overall enrichment being weaker in MII, zygotes, and 2-cell embryos than in GV, 4-cell, 8-cell, and blastocyst stages (Liu et al., 2025; Hu, 2022). In distal regions, a progressive erasure from the zygote culminated in the establishment of a stable pattern after the 4-cell stage, which was then maintained through the blastocyst (Liu et al., 2025; Hu, 2022) (Figure 3).

H3K4me1 exhibits dynamic changes during early embryonic development, indicating that its deposition is under precise spatiotemporal control. To elucidate the regulatory mechanisms and functions of this histone mark, the following section will focus on the key enzymes responsible for catalyzing H3K4me1 and their roles in development. In 2013, MLL3 and MLL4 were first identified in cell lines as the primary H3K4me1 methyltransferases at enhancers (Hu et al., 2013; Lee et al., 2013). Subsequent functional studies in early mouse embryos revealed that inhibiting H3K4 methylation impairs minor zygotic genome activation on the paternal genome, leading to developmental arrest, underscoring a critical role for MLL3/4 in preimplantation development (Aoshima et al., 2015). This functional requirement is further specified by recent findings that the methyltransferase activities of MLL3/4 exert lineage-selective functions during early embryogenesis and ESC differentiation (Xie et al., 2023). However, work by Kubo, N. et al. in neural differentiating mESCs demonstrates that in the absence of MLL3/4 catalytic activity, KMT2B can partially compensate by depositing H3K4me1 at enhancer regions, maintaining a subset of enhancer-promoter interactions and supporting transcriptional activation (Kubo et al., 2024). Collectively, these findings highlight the robustness and complexity of the epigenetic regulatory network during embryonic development.

### 3.2 Histone acetylation

Histone acetylation serves as a highly dynamic and critical epigenetic regulatory mechanism during early embryonic development. During key windows of mammalian embryonic development, the balance between histone acetyltransferases (HATs) and deacetylases (HDACs) enables rapid and precise response to external signals, ensuring timely regulation of developmental genes (Bozdemir and Uysal, 2023). Notably, previous studies have established that the HATs activity of p300 catalyzes the acetylation of multiple lysine residues on histone tails, including H3K27, H3K9 (Zhang et al., 2018).

#### 3.2.1 H3K27ac

The histone mark H3K27ac, deposited by CBP/p300 acetyltransferases (Stasevich et al., 2014), serves as a critical regulator of active enhancers and promoters, exhibiting strong correlation with transcriptional activity (Zhang et al., 2022). It distinguishes active enhancers from poised enhancers (marked by H3K4me1 (Creyghton et al., 2010) or H3K27me3 (Rada-Iglesias et al., 2010)). Wang, M. *et al.* demonstrated, through combined immunofluorescence and sequencing analyses, that H3K27ac undergoes three major global transitions during maternal-to-zygotic transition in mice, accompanied by the emergence of narrow peaks at the 2-cell stage (Wang et al., 2022). During early human development, H3K27ac also undergoes extensive remodeling: pre-ZGA embryos exhibit broad H3K27ac domains overlapping with broad H3K4me3, which resolve into sharp peaks post-ZGA that are linked to stage-specific gene expression (Wu K. et al., 2023). Recently, Liu, B. *et al.* characterized the dynamic changes of H3K27ac during oogenesis and early embryogenesis, they found that embryo-specific enhancers are primed before ZGA and identified TCF3/12 as key regulators of folliculogenesis (Liu et al., 2024). Xiang Y.L. et al. systematically mapped the H3K27ac modification landscape in the mouse gastrula stage and revealed that the ectoderm, mesoderm, and endoderm each possess lineage-specific putative enhancers (Xiang et al., 2020). Theses studies indicated the dynamic changes in H3K27ac may serve as a key epigenetic regulator in cell fate determination. Furthermore, although the narrowing of broad H3K27ac domains after ZGA is proposed to be driven by HDAC-mediated deacetylation, the potential contributions of other mechanisms-such as changes in transcription factor binding specificity-remain to be fully elucidated (Figure 3).

#### 3.2.2 H3K9ac

H3K9 acetylation is primarily catalyzed by the GCN5/PCAF complex, rather than by the general coactivators CBP/p300 (Jin et al., 2010). It co-localizes with H3K4me3 at active gene promoters, where H3K4me3 facilitates transcription initiation, while H3K9ac promotes RNA polymerase II pause-release by recruiting the super elongation complex (SEC) to chromatin (Gates et al., 2017). In mouse early embryos, H3K9ac accumulates at retrotransposons, while H3K27ac appear to assume distinct functions in their activation (Wu K. et al., 2023). Furthermore, in SCNT embryos, aberrant H3K9ac disrupts genome activation at the 2-cell stage, a deficiency rescued by Dux overexpression (Yang et al., 2020). This study applied ultra-low-input nucleosome ChIP sequencing (ULI-NChIP-seq) to generate comprehensive genome-wide profiles of H3K9ac in mouse naturally fertilized (NF) and SCNT embryos at the 1-cell (1C), 2-cell (2C), and morula stages (Yang et al., 2020). During neural differentiation of hESCs, H3K9ac exhibits a biphasic dynamic: it decreases initially (days 0–4) to silence pluripotency genes, then increases (days 4–8) to activate neural developmental genes (Qiao et al., 2014). Collectively, these findings underscore that H3K9ac modulates embryonic development through stage- and context-dependent gene regulation.

### 3.3 Histone ubiquitination

**FIGURE 4:**
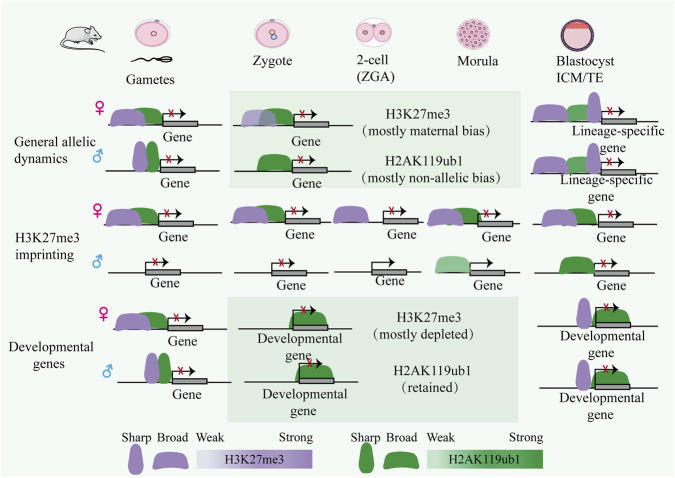
The genomic landscape of H3K27me3 and H2AK119ub1 co-localization. These models illustrate the dynamic changes of H3K27me3 and H2AK119ub1 from gametes to blastocysts in mouse early embryos, particularly in contexts of H3K27me3 imprinting and developmental gene silencing. Within these regions, the two histone modifications exhibit substantial colocalization. Notably, H3K27me3 maintains maternal-biased domains throughout preimplantation development, whereas H2AK119ub1 distribution becomes largely balanced by the two-cell stage (Chen et al., 2021; Mei et al., 2021; Zhang et al., 2016). At H3K27me3 imprinted loci, maternal H3K27me3 domains persist through preimplantation development (Zhang et al., 2016); however, H2AK119ub1 at these sites is transiently lost at the two-cell stage and is subsequently reestablished in a locus-specific manner during later stages (Chen et al., 2021; Mei et al., 2021). The transcriptional silencing of developmental genes in preimplantation embryos does not strictly depend on H3K27me3, a phenomenon that may be explained by the sustained presence of H2AK119ub1.

In early embryos, histone ubiquitination exerts context-dependent functions, in a locus-specific manner to instruct cell fate transitions. The repressive mark H2AK119ub1, dynamically regulated by PRC1 and the Polycomb repressive deubiquitinase complex (PR-DUB), plays a pivotal role in embryogenesis. In mouse embryos, H2AK119ub1 and H3K27me3 exhibit partial genomic overlap yet maintain functional autonomy, however, loss of H2AK119ub1 results in premature activation of developmental genes during ZGA (Chen et al., 2021) (Figure 4). It is noteworthy that H3K27me3 maintains a maternally biased distribution until the blastocyst stage, whereas H2AK119ub1 is preferentially enriched on the maternal genome in the zygote and becomes evenly distributed between parental genomes by the two-cell stage (Chen et al., 2021; Mei et al., 2021; Zhu et al., 2021). During ZGA, H2AK119ub1 is deposited at Polycomb targets prior to H3K27me3, where it guides the establishment and embryonic inheritance of maternal H3K27me3 (Mei et al., 2021). Throughout subsequent developmental stages, repressive histone modifications at PcG (Polycomb group proteins) target loci exhibit distinct dynamics, largely following two divergent trajectories (Chen et al., 2021; Mei et al., 2021; Zhu et al., 2021). At the majority of loci, H2AK119ub1 persists during preimplantation development, whereas H3K27me3 is gradually erased after fertilization and is only fully restored by the epiblast stage (Chen et al., 2021; Mei et al., 2021; Zhu et al., 2021). In contrast, a subset of targets escapes this reprogramming and retains both H3K27me3 and H2AK119ub1 concurrently throughout the entire preimplantation period (Chen et al., 2021; Mei et al., 2021; Zhu et al., 2021). Interestingly, USP16, the major deubiquitinase in mouse oocytes, rapidly clears maternal H2AK119ub1 to permit ZGA (Rong et al., 2022). Its loss leads to embryonic arrest at the 2-cell stage and reduced developmental potential (Rong et al., 2022). Similarly, USP7 depletion disrupts morula-to-blastocyst transition and dysregulates lineage-specifying genes (e.g., *Cdx2, Oct4, Nanog, Sox2*) (Yu et al., 2024). DNMT3A also recognizes H2AK119ub1-marked nucleosomes *via* a ubiquitin-dependent recruitment (UDR) domain to promote DNA methylation and genomic stability (Chen et al., 2024). While direct human data are limited, functional conservation is likely. In summary, a growing body of evidence reveals that the dynamic landscape of H2AK119ub1 likely constitutes a fundamental regulatory layer underlying cell fate transitions during early embryogenesis.

### 3.4 Metabolically-associated histone modifications: the emerging roles of crotonylation and lactylation

Recent advances in epigenetics have uncovered critical roles of novel histone modifications, including crotonylation (Tan et al., 2011), and lactylation (Zhang et al., 2019), in orchestrating cell fate determination during early embryogenesis. These modifications dynamically remodel chromatin architecture and recruit lineage-specific transcriptional regulators.

**FIGURE 5:**
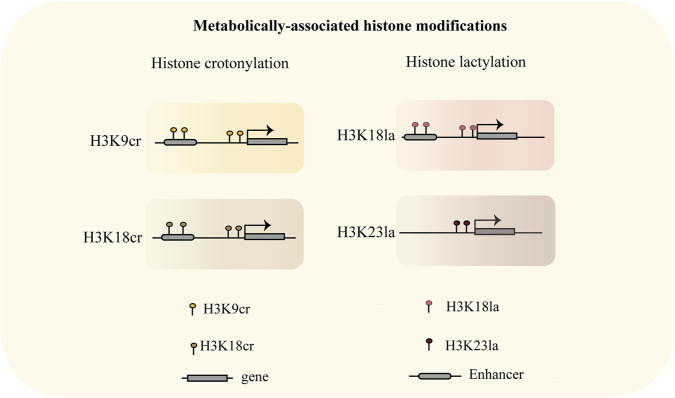
Genomic localization and transcription al regulatory roles of metabolically-linked histone modifications. H3K9cr, H3K18cr, and H3K18la are present at both promoter and distal enhancer regions, where they promote transcriptional activation (Gao et al., 2024; Li J. et al., 2023). H3K23la is enriched at promoter regions and functions in transcriptional activation (Yang et al., 2021).

Histone crotonylation (Kcr), is catalyzed by the known histone acetyltransferases such as p300/CBP and MOF (Liu et al., 2017), while Class I HDACs have been identified as major decrotonylases (Wei et al., 2017). Studies indicate that Kcr acts as a metabolically sensitive mark that promotes endoderm differentiation in human ESCs (Fang et al., 2021). Further investigation reveals that this process involves extends beyond histone modification to include crotonylation of non-histone proteins such as GAPDH, coupled with a metabolic shift from glycolysis to oxidative phosphorylation (Zhang et al., 2023). Notably, Kcr such as H3K9cr are enriched at highly active promoter regions, where they enhance chromatin accessibility, recruit RNA polymerase II, and facilitate the activation of bivalent promoters, thereby contributing to neural stem cell fate determination (Dai et al., 2021) (Figure 5). To further explore the dynamics and function of histone crotonylation during early embryonic development, Gao, D.*, et al.* demonstrated that p300 knockdown significantly reduces H3K18cr and H3K9cr levels, leading to downregulation of key lineage-specific genes (including *Oct4*, *Gata3,* and *Nanog*) and developmental arrest at the 8-cell stage (Gao et al., 2024). Wang, Y.-F. *et al.* Further delineated the genome-wide landscape of histone crotonylation in mouse and human early embryos, revealing that Kcr promotes zygotic genome activation and repetitive element expression during mammalian preimplantation development (Wang Y.-F. et al., 2025). Thus, Kcr is precisely regulated during early embryogenesis, where it coordinates cell fate decisions by modulating chromatin state, gene expression, and metabolic reprogramming.

Histone lactylation, a lactate-derived post-translational modification, functions as both a metabolic sensor and epigenetic regulator that directly links glycolytic activity to developmental gene transcription (Merkuri et al., 2024; Zhang et al., 2019). In mammals, H3K18la, one of the major forms of histone lactylation, serves as a mark of both active promoters and tissue-specific enhancers (Galle et al., 2022) (Figure 5). Further studies have delineated the landscape of H3K18la in mouse and human preimplantation embryos and demonstrated its essential role during major ZGA (Li J. et al., 2023). Complementary work has elucidated the functional contribution of histone lactylation to oocyte maturation and embryogenesis (Yang et al., 2024). Concurrently, systematic profiling of H3K23la, H3K18la, and pan-histone lactylation in mouse oocytes and preimplantation embryos revealed that hypoxic *in vitro* culture conditions reduce global histone lactylation and impair developmental progression (Yang et al., 2021). Collectively, these findings establish histone lactylation as a pivotal metabolite-sensitive epigenetic modification that plays a fundamental role in coordinating metabolic state with gene regulatory networks during early mammalian development.

In summary, the dynamic regulation of these newly characterized epigenetic modifications critically influences the ZGA and lineage specification in mouse embryogenesis.

### 3.5 Histone variants

Epigenetic reprogramming in the early embryo also involves the incorporation of histone variants, such as H3.3 and H2A.Z, which replace canonical histones in nucleosomes and modify chromatin structure and gene expression (Lin et al., 2013; Liu X. et al., 2022). In preimplantation mouse embryos, H3.3 plays a crucial role in supporting normal development by maintaining a proper chromatin condensation (Lin et al., 2013). Recent study delineating of the H3.3 landscapes in mouse oocytes and embryos showed that, while H3.3 is relatively uniformly distributed in a non-canonical manner in mature oocytes and zygotes, it switches to a canonical pattern by the 2-cell stage in a replication-coupled manner (Ishiuchi et al., 2020). Meanwhile, H2A.Z is essential for oocyte maturation, proper meiotic progression, and fertility in female mice (Mei et al., 2025; Xu Q. et al., 2025). During mouse oocyte growth, H2A.Z gradually shifts from a canonical distribution, enriched at active promoters, to a non-canonical broad distribution (ncH2A.Z) (Mei et al., 2025). In mouse fully grown oocytes (FGOs), H2A.Z forms broad domains across intergenic regions, co-localizing with non-canonical H3K4me3 (ncH3K4me3), and becomes depleted from CpG-rich silent promoters (Mei et al., 2025; Xu Q. et al., 2025). H2A.Z landscapes in mouse early embryos showed that paternal H2A.Z is removed upon fertilization, followed by unbiased accumulation on parental genomes during ZGA (Liu X. et al., 2022). Notably, H2A.Z shows promoter-type-specific hierarchical deposition: double peaks with H3K4me3 indicate activation; single peaks bearing the H3K4me3/H3K27me3 bivalent mark suggest repression of developmental genes; and H2A.Z-free promoters remain silenced in early embryos (Liu X. et al., 2022).

## 4 Chromatin accessibility

Chromatin accessibility, reflecting the degree to which genomic DNA is available for interaction with regulatory proteins, serves as a fundamental determinant of cellular identity and developmental potential. The dynamic interplay between chromatin openness and epigenetic modifications (including DNA methylation and histone marks) creates a precisely regulated landscape that guides embryonic development (Atlasi and Stunnenberg, 2017; Wilkinson et al., 2023).

Research in mouse embryos describes multifaceted SOX2-enhancer interactions during pluripotency transition (Li L. et al., 2023). This work conceptualizes SOX2 binding into distinct modes: settler binding in the E3.5 ICM, where SOX2 occupies enhancers pre-opened by other factors and its loss does not substantially alter chromatin accessibility; pioneer binding in the E4.5 epiblast or 2i mESCs, where SOX2 is necessary to establish or maintain enhancer accessibility; and pilot binding in 2i mESCs, which may help poise enhancers for faster opening (Li L. et al., 2023). Concurrently, in human 4-cell embryos, maternal OTX2 directly targets and opens chromatin at critical regulatory elements of EGA genes (Wang Q. et al., 2025). These findings collectively illustrate how specific transcription factors can shape unique chromatin accessibility landscapes at precise developmental times. Notably, the evolution of chromatin accessibility profiling—from early DNase I hypersensitivity mapping to spatial ATAC-seq—has revolutionized our ability to dissect cell fate decisions in early mouse and human embryos with unprecedented resolution (Buenrostro et al., 2013; Jin et al., 2015; Sun et al., 2024). The recently developed scNanoATAC-seq2 technique employs long-read sequencing to enable high-resolution mapping of chromatin accessibility in early mouse embryos at single-cell resolution (Li M. et al., 2025).

The developmental significance of chromatin accessibility is particularly evident during critical transitions. Studies of the accessible chromatin landscape in mouse preimplantation embryos reveal that its dynamic remodeling is profoundly shaped by transposable elements and is closely associated with an extensive repertoire of putative cis-regulatory sequences (Wu et al., 2016). Following fertilization, maternal mRNAs and proteins are progressively degraded, while the zygotic genome becomes activated. During human early embryonic development, numerous chromatin regions that were initially accessible undergo closure after ZGA. And this transition is closely linked to the reprogramming of a non-canonical form of H3K4me3 (Wu et al., 2018). Apparently, in humans, the paternal genome exhibits higher chromatin openness than the maternal genome as early as the metaphase stage of the zygote, and this differential accessibility persists until the 4-cell stage (Li et al., 2018). Multi-omics approaches have demonstrated how accessible regions interact with cis-regulatory elements and transposable elements to form functional gene regulatory networks (GRNs) that drive lineage segregation (Gao et al., 2018; Jiang et al., 2023; Wu et al., 2018; Xu et al., 2024). The functional consequences of aberrant chromatin accessibility are profound, as exemplified by Tgfbr1-deficient mouse embryos (Lozovska et al., 2024; Reardon, 2024). Future studies integrating high-resolution multi-omics approaches will further elucidate how accessibility dynamics interface with other epigenetic layers to orchestrate developmental transitions.

## 5 3D remodeling

Chromatin is not linearly and randomly folded, but rather organized into specific high-order architectures through multi-level folding. The tight or loose configuration of nucleosomes forms the structural basis of this three-dimensional organization (Wen et al., 2025). Beyond this fundamental level, the 3D genome is organized into higher-order structures including topologically associating domains (TADs) (Okhovat et al., 2023), compartment A/B (Harris et al., 2023), and chromatin loops (Grubert et al., 2020). The assembly, disassembly, and transition of these hierarchical structures enable spatiotemporal regulation of genes critical to cell fate. Similarly, the dynamic remodeling of these structures is intimately linked to histone modifications, gene expression, and chromatin accessibility, collectively establishing the foundation for subsequent embryonic development and lineage commitment (Bai et al., 2025; Collombet et al., 2020; Du et al., 2019).

**FIGURE 6:**
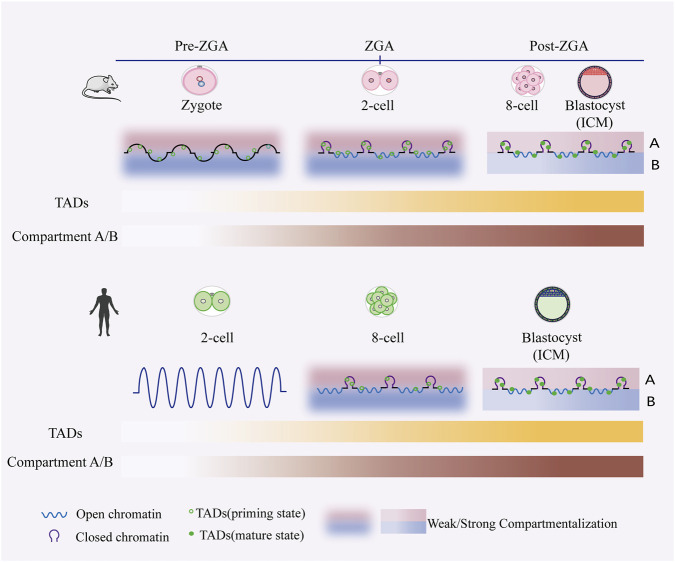
Epigenetic reprogramming of 3D chromatin architecture in mouse and human early embryos. In mouse embryos, higher-order chromatin structures are not yet prominent during the zygotic and ZGA stages, though spatial segregation and compartmentalization are already detectable (Du et al., 2017; Ke et al., 2017). In contrast, human embryos exhibit relatively uniform chromatin distribution without clear compartmentalization at the 2-cell stage (Chen et al., 2019). Throughout preimplantation development in both species, higher-order chromatin architecture is progressively established, marked by the gradual consolidation of topologically associating domains (TADs) and A/B compartments.

In mouse embryos, TADs are almost unstructured in zygotes and two-cell embryo, and the re-establishment of higher-order chromatin architecture is DNA replication-dependent and occurs gradually during embryogenesis (Du et al., 2017; Ke et al., 2017) (Figure 6). Subsequent investigations of higher-order chromatin structure in the parental genomes after fertilization revealed that it correlated with allelic-specific enrichment of H3K27 methylation and contributed to parentally biased gene expression (Collombet et al., 2020). Concurrently, a self-interacting, cohesin-independent compartmentalized domain has been identified in mouse oocytes (Du et al., 2019). These domains, marked by H3K27me3, are termed Polycomb-associated domains (PADs) (Du et al., 2019). However, TAD boundaries are highly conserved across species (Okhovat et al., 2023). Demarcated by insulator elements such as CTCF, cohesin complexes (Dixon et al., 2012), these boundaries confine chromatin contacts within specific domains and serve as central regulators of gene expression during early embryogenesis (Dong et al., 2024; Wang W. et al., 2024). CTCF does not function in isolation within the gene regulatory network. Instead, it exhibits a delicate antagonistic interplay with DNA methylation, which plays a critical role in regulating genes and orchestrating cell fate determination during ESC differentiation (Monteagudo-Sánchez et al., 2024). CTCF also orchestrates the gene expression changes within metabolic and proteostatic programs that occur during the morula-to-blastocyst transition in mouse embryos (Andreu et al., 2022). Unexpectedly, recent studies have revealed that cohesin, rather than CTCF, exhibits a stronger temporal correlation with the re-establishment of topologically associating domains (TADs) (Yu et al., 2025).

Furthermore, TADs constitute the structural foundation of higher-order chromatin organizations known as compartment A/B. During embryogenesis, compartment A is generally associated with transcriptional activity, while compartment B is linked to gene repression (Lieberman-Aiden et al., 2009). It is noteworthy that compartment A/B in human embryos experiences dynamic disappearance and reconstruction (Chen et al., 2019). Their interconversion potentially represents a critical epigenetic hallmark signifying cell fate commitment (Chu and Wang, 2020). The presence of TADs and loops, but not compartments, in the maternally inherited zygotic chromatin suggests that compartments are established by a distinct, independent mechanism (Flyamer et al., 2017). Simultaneously, within TADs, the loop extrusion activity of the cohesin complex brings distal enhancers into precise spatial proximity with target gene promoters, forming specific “point-to-point” contact structures known as chromatin loops (Davidson et al., 2019; Han et al., 2025). The cohesin complex, in concert with CTCF, orchestrates long-range chromatin looping associated with domain architecture, whereas ZNF143 and HCFC1 mediate the formation of short-range loops that directly regulate gene expression (Liu S. et al., 2022).

In summary, the multilevel architecture of chromatin—from the insulating properties of topologically associating domains (TADs), through the large-scale dynamics of A/B compartments, to the precise enhancer-promoter interactions mediated by chromatin loops—collectively demonstrates a tight coupling with transcriptional regulation.

## 6 Discussions

Epigenetic reprogramming during early embryonic development is a highly intricate and precisely regulated biological process. It encompasses global erasure of DNA methylation, dynamic changes in histone modifications, regulation of chromatin accessibility, and remodeling of the three-dimensional chromatin architecture. At the level of histone modifications, these can be further categorized into methylation, acetylation, ubiquitination, metabolically-associated modifications such as crotonylation, and lactylation and histone variants. Notably, histone methylation itself can be subdivided based on functional roles and genomic localization, including gene activation-associated H3K4me3, facultative heterochromatin marker H3K27me3, bivalent modifications (H3K4me3 and H3K27me3) with dual activation and repression potential, constitutive heterochromatin mark H3K9me3, gene body-enriched H3K36me3, and the enhancer-specific modification H3K4me1. Through a systematic review and synthesis of recent studies, several key conclusions can be drawn.

During early embryonic development, DNA methylation undergoes genome-wide establishment and erasure to guide the resetting of cellular identity during reprogramming. This process is dynamically regulated by DNA methyltransferases (writers) and demethylases (erasers), which collectively ensure precise control over cell fate transitions. At the level of histone modifications, H3K4me3 transitions from a broad, non-canonical distribution to a sharp, canonical peak pattern, ensuring timely activation of lineage-specifying genes following ZGA (Zhang J. et al., 2025). After fertilization, H3K27me3 is markedly lost at promoters of developmental regulators such as Hox genes, while distal H3K27me3 domains inherited from the oocyte are retained (Zhang et al., 2016). The establishment and dynamic resolution of bivalent chromatin domains—marked by the coexistence of H3K4me3 and H3K27me3—represent a key mechanism in maintaining pluripotency and lineage plasticity. Meanwhile, H3K9me3 exhibits stage-specific distribution and plays context-dependent roles during development (Wang C. et al., 2018). H3K36me3, on the other hand, contributes to the fidelity of transcription initiation and elongation (Neri et al., 2017). The enhancer-associated mark H3K4me1 also undergoes dynamic remodeling in early embryos. Notably, a transition from H3K27me3 to H3K4me1 is observed at bivalent promoters, a process critical for subsequent lineage specification (Yu et al., 2023).

During ZGA, the deposition of H3K27ac occurs prior to the onset of gene transcription, and its modification levels are finely tuned by the coordinated actions of CBP/p300 and HDACs. Additionally, the loss of H2AK119ub1 leads to premature activation of developmental genes at the ZGA stage (Chen et al., 2021). The recently characterized histone lactylation and crotonylation modifications provide novel insights into metabolic regulation of development, directly coupling glycolytic flux with chromatin states to influence pluripotency and lineage commitment (Fang et al., 2021; Wu H. et al., 2023). Notably, Chromatin accessibility dynamics frequently precede transcriptional changes, with intercellular heterogeneity in open chromatin regions potentially establishing fate flexibility. Similarly, the reorganization of 3D chromatin architecture occurs prior to morphological differentiation, and its conformational transitions may presage future fate decisions. The impact of epigenetic remodeling on embryonic fate transitions is manifested in three major aspects. First, it regulates the timing and pattern of ZGA, ensuring a smooth transition from maternal to zygotic control. Second, it directs the proper differentiation of the ICM and TE by establishing and maintaining lineage-specific epigenetic landscapes. Third, dysregulation of this reprogramming can lead to severe developmental defects, including embryonic arrest.

However, previous studies predominantly focused on averaged modification profiles at the whole-embryo or bulk level, which limited the resolution to capture cell-to-cell heterogeneity and precise correlation between histone modification dynamics and lineage specification. The scMTR-seq technique pioneers the simultaneous mapping of six histone modifications combined with transcriptome sequencing (Wang Y. et al., 2025). Meanwhile, TACIT/CoTACIT enables genome-wide profiling of seven histone modifications in individual cells (Liu et al., 2025), while scNanoHi-C2 captures the dynamic reprogramming of 3D genome architecture in mouse embryonic-stage germ cells (Lu J. et al., 2025). These methods hold great promise for overcoming the technical challenges of working with rare and heterogeneous embryonic samples. Although the application of single-cell technologies has significantly advanced our understanding of epigenetic dynamics during embryogenesis, many questions remain unresolved. For instance, the dynamic distribution of H3K4me1 in sperm is still not fully characterized, and whether emerging modifications such as histone crotonylation and lactylation exhibit non-canonical modes remains to be explored. Furthermore, due to ethical constraints and technical limitations, the precise functions and regulatory networks of these modifications in human embryos await systematic elucidation.

Looking forward, epigenetic research in early embryonic development is poised to advance along two major frontiers. First, while current studies have predominantly focused on pre-implantation embryos, the peri-implantation period, encompassing the late blastocyst to early gastrulation stages, remains largely unexplored in terms of epigenetic reprogramming. This critical window for cell fate determination and lineage specification presents a significant knowledge gap due to challenges in sample acquisition and technical limitations. Utilizing embryo-like models for controlled experimentation and comparative analysis holds promise for systematically uncovering how epigenetic remodeling drives developmental fate decisions. Second, the ongoing integration of advanced methodologies, particularly combining single-cell multi-omics with CRISPR-Cas9 screening tools, will provide powerful means to decipher the combinatorial regulatory rules of epigenetic modifications. A particularly promising direction is elucidating how metabolic pathways guide epigenetic remodeling and regulate embryonic development, which is expected to become a key frontier in developmental biology. Progress along these research avenues will collectively contribute to a dynamic, multiscale understanding of early development, from molecular and cellular processes to structure and function.
